# O.4.1-3 Effects of novel isometric resistance training on resting and ambulatory blood pressure measures: a comparison between exercise modes

**DOI:** 10.1093/eurpub/ckad133.168

**Published:** 2023-09-11

**Authors:** Ben Henry Wright, Mark Robert Antrobus, Peter Gordon Woodrow Jones, Anthony William Baross

**Affiliations:** University of Northampton, UK; ben.wright@northampton.ac.uk; University of Northampton, UK; ben.wright@northampton.ac.uk; University of Northampton, UK; ben.wright@northampton.ac.uk; University of Northampton, UK; ben.wright@northampton.ac.uk

## Abstract

**Purpose:**

Isometric resistance training (IRT) can reduce clinical resting, and ambulatory blood pressure measures (ABPM) irrespective of hypertensive status, yet adopted methods are expensive, require specialised equipment, and may limit accessibility for those with mobility restrictions. Previous work undertaken in our laboratory has established a novel isometric training band (ITB) and associated protocol as a method of safely performing acute IRT. However, the chronic effects of ITB on clinical resting blood pressure (BP) and ABPM remains unknown. Therefore, the aim of this study was to validate the novel IRT method by comparing the effects of the ITB on BP following 4-weeks of IRT.

**Methods:**

Forty-two healthy normotensive adults (22 male, 20 female, [Mean ± SD], age 31 ± 14 years, height 170 ± 10 cm, mass 75 ± 16 kg, systolic [SBP], 120 ± 5 mmHg, diastolic [DBP], 72 ± 7 mmHg, mean arterial [MAP], 88 ± 6 mmHg, heart rate [HR[ 67 ± 12 bpm·^-1^) were randomised to a control (CON), isometric handgrip (IHG) or isometric training band (ITB) group. Clinical resting (SBP, DBP, MAP, & HR) and ABPM (n = 38; 24-hour, daytime, and night-time SBP, DBP, MAP, & HR) were measured pre-and post-4-weeks of supervised IRT (4 x 2-minute contractions at 30%MVC [IHG] or 4 x 2-minute contractions at CR-10 values equivalent to 30%MVC [ITB]). Data were analysed using two-way repeated measures ANOVAs to examine any significant (*P* <.05) within-and between group differences.

**Results:**

Clinical resting SBP was reduced for IHG (-4.6 ± 3.6 mmHg, *P* <.05) and ITB (-4.5 ± 3 mmHg, *P* <.05) following 4-weeks of IRT. No differences were seen within-or between groups for clinical resting DBP and MAP or for resting HR (*P* >.05). No differences were found over time or between groups for ABPM (*P* >.05 across all measures).

**Conclusions:**

These findings suggest 4-weeks of IRT using the novel ITB can elicit reductions in clinical resting BP in normotensives comparable to IHG. As such, the ITB may offer a practical and cost-effective alternative to long-established IRT methods. Yet further research is needed to ascertain the ABPM-lowering effects of the ITB for normotensive groups.

